# Phenotip - a web-based instrument to help diagnosing fetal syndromes antenatally

**DOI:** 10.1186/s13023-014-0204-7

**Published:** 2014-12-10

**Authors:** Shay Porat, Maud de Rham, Davide Giamboni, Tim Van Mieghem, David Baud

**Affiliations:** Department of Obstetrics and Gynaecology, Hadassah University Hospital, Mount Scopus campus, Jerusalem, Israel; Materno-Fetal and Obstetrics Research Unit, Department of Obstetrics and Gynaecology, University Hospital, Lausanne, Switzerland; TechCare.ch, Geneva, Switzerland; Department of Obstetrics and Gynaecology, Division of Woman and Child, University Hospitals Leuven, Leuven, Belgium

**Keywords:** Syndrome, Prenatal ultrasound, Free web-based tool, Sonographic markers

## Abstract

Prenatal ultrasound can often reliably distinguish fetal anatomic anomalies, particularly in the hands of an experienced ultrasonographer. Given the large number of existing syndromes and the significant overlap in prenatal findings, antenatal differentiation for syndrome diagnosis is difficult. We constructed a hierarchic tree of 1140 sonographic markers and submarkers, organized per organ system. Subsequently, a database of prenatally diagnosable syndromes was built. An internet-based search engine was then designed to search the syndrome database based on a single or multiple sonographic markers. Future developments will include a database with magnetic resonance imaging findings as well as further refinements in the search engine to allow prioritization based on incidence of syndromes and markers.

## Findings

### Background

Many countries have incorporated ultrasound in routine prenatal care for fetal anomaly screening. When multiple fetal anomalies are found, a syndrome is often suspected. Some syndromes have a known genetic background and can be identified by invasive fetal testing with routine karyotyping and/or comparative genomic hybridization (e.g. Edwards syndrome or DiGeorge syndrome). Many others however, require specific gene sequencing or do not have a known genetic origin (such as Noonan syndrome or Fryns syndrome) and cannot be identified by routine genetic screening tests. Accurate prenatal identification or suspicion of a syndrome is therefore important to guide further testing and/or counseling. Given the large number of known syndromes [1] (over 6000) and a significant overlap in prenatal findings, antenatal differentiation is difficult. The OMIM® (Online Mendelian Inheritance in Man) database [2], Orphanet [3], Possumweb [4] and London Medical Database [5] are searchable databases that allow links of phenotypic findings with (genetic) syndromes and may help in diagnosing syndromes. None of the database queries, however, include prenatal ultrasound findings (such as echogenic bowel or increased nuchal fold) in the search algorithm. Moreover, as these databases are mainly designed for postnatal use, they give great importance to markers that may not always be present or identifiable in the prenatal stage (such as failure to thrive, microcephaly or neurodevelopmental delay). Finally, these databases deal poorly with marker synonyms. As an example, the search terms “*echogenic kidneys*” and “*hyperechogenic kidneys*” yield 15 and 18 syndromes respectively in OMIM® [2], but only three syndromes are shared by both searches.

The need for a freely available tool, useable in the prenatal period, brought us to design ‘Phenotip’, a free web-based searchable syndrome database, which is based exclusively on sonographic markers.

## Methods

### Database design

The Phenotip collaboration is an independent, international association between maternal-fetal medicine specialists with particular interest in prenatal diagnosis and a software engineer. The Phenotip database relies on a hierarchically structured “tree” of antenatal sonographic markers (n = 1140). Parent markers are organized by organ system and grow in resolution with every level of branching (daughter markers). For example, “face” branches into “eyes”, “ears”, “mouth and lips”. “Mouth and lips” then further branches into “lip”, “palate”, “philtrum” and so on. Therefore, each marker has multiple parent and/or daughter markers. Marker synonyms have been defined to avoid confusion (e.g. talipes – clubfoot). Overall, 1140 sonographic markers are available, among them 130 markers have at least one synonym.

Markers are grouped into syndromes based on an extensive literature search. Only markers that were previously described in a peer reviewed publication as part of the antenatal sonographic phenotype of a proven syndrome were included in the database. Each syndrome is defined by its specific daughter markers, but also includes all hierarchically superior parent markers.

When this information was available, we also noted the incidence and inheritance pattern and male/female ratio for each syndrome. Weblinks to relevant overview articles or websites such as OMIM*®* [2], Orphanet [3], Geneva Foundation [6], Jablonski’s database [7] and SonoWorld [8] were added.

Information for each syndrome was registered by one editor, then peer-reviewed by at least one other editor. So far, we have collected literature on 329 of the most common syndromes.

### Searching the database

The syndrome database is freely available through a web-based interface at www.phenotip.com. Users can search by syndrome name or by a combination of ultrasound markers.

When a specific marker is chosen, the search algorithm automatically includes all daughter markers of the chosen marker. Each level of the hierarchical tree of each specific organ system is thus considered. Choosing a parent marker will increase the sensitivity of the search while choosing a daughter marker will increase specificity. When a sonographic abnormality is not clearly defined, the involved organ can be selected, and hence all downstream markers would be considered. This is, for example, useful in cases of cardiac malformations, where one syndrome may present with a wide variety of heart lesions. Also, non-experienced sonographers might select the affected organ when they are unable to define the exact cardiac pathology.

Markers can either be selected from an expandable hierarchic tree or from a search box. Users can choose to search only syndromes including “all selected markers” or to search syndromes including either “one of the selected markers”, thereby again increasing sensitivity or specificity, respectively.

## Results

**Figure 1:**
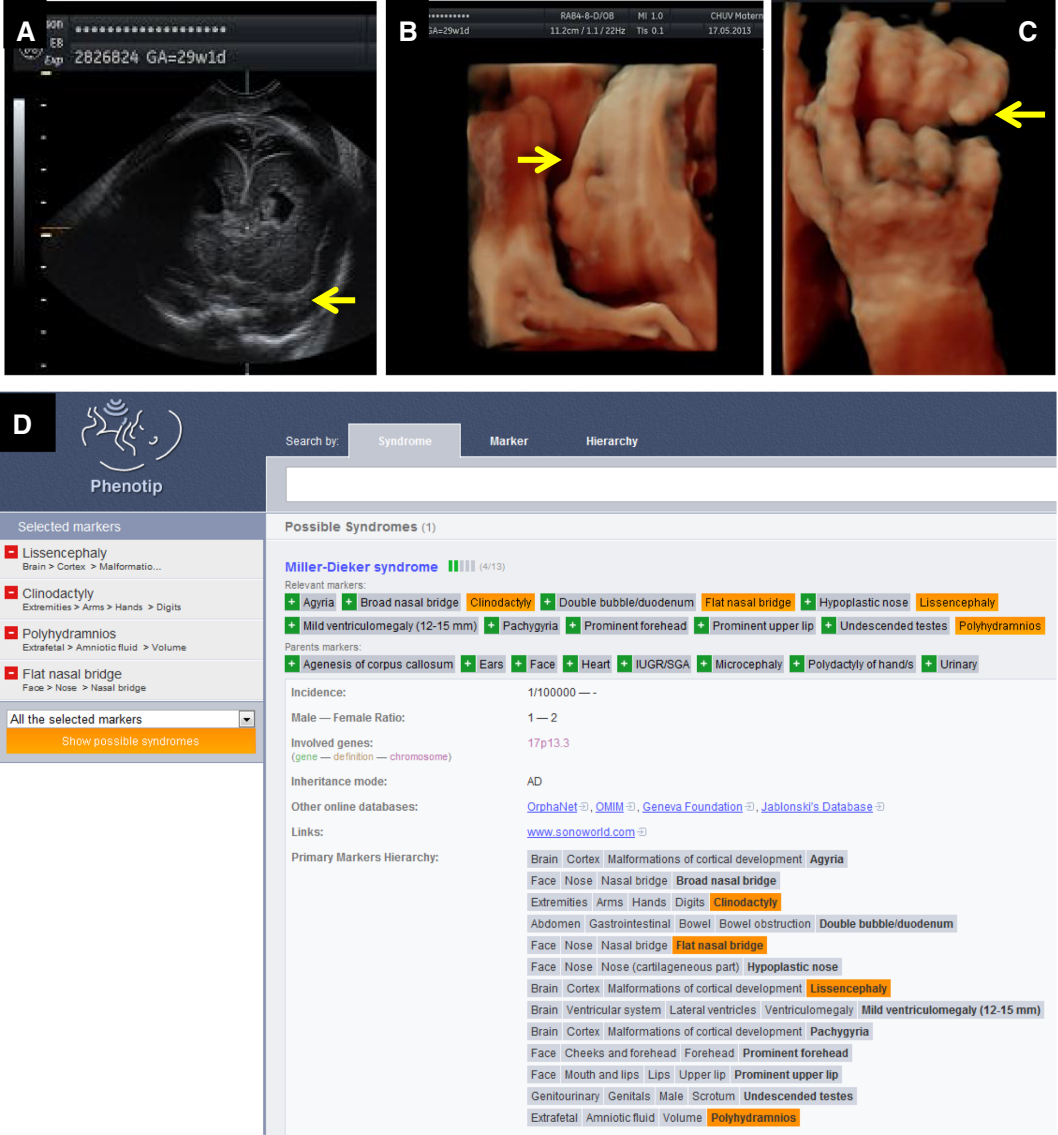
**A 28 years old primigravida patient was admitted at 29 weeks gestation for short cervix and abdominal pain.** Serologies, 1^st^ trimester screening and anatomy ultrasound at 20 weeks were all normal. On admission, ultrasound showed *polyhydramnios*, *lissencephaly* (**A**, coronal view), *flat nasal bridge* (**B**, sagittal 3D-view of the face) and *clinodactyly* (**C**, 3D-view of the hands). All images kindly provided by Yvan Vial, Lausanne-CHUV, Switzerland. Using the Phenotip.com database, these markers were suspected for a Miller-Dieker syndrome (**D**). In the Phenotip website, markers can be searched through a hierarchy tree (top right) or the marker search box (top middle). Each selected marker will appear on the left hand side of the screen under “selected marker” after clicking the green button. They can also be removed by clicking the red button. Differential diagnosis will appear after clicking the “show possible syndromes” button on the left hand side of the screen. Amniodrainage was performed, and CGH array confirmed a micro-deletion of locus p13.3 on chromosome 17 including LIS1gene.

Since its inception in July 2013, the Phenotip database has logged 1215 sessions by 714 users with, among them, 136 regular visitors from 18 countries. The tool has allowed the identification of a sometimes-unsuspected diagnosis in many cases. A recent example suspected through our search algorithm and then confirmed by genetic analysis is presented in Figure 1.

Phenotip allows the search of differential diagnosis either by replacing a specific marker (“Flat nasal bridge”) by a less specific marker (“Face”), or by removing a marker from the searching list. Table 1 provides an example based on markers used in Figure 1 (Miller-Dieker syndrome).

**Table 1:** **Differential diagnosis found using 4, 3 and 2 markers from Figure** 1

**Markers inserted in Phenotip.com**	**Number of/diagnosis found with Phenotip.com (except Miller-Dieker Sd)**
**4 markers**	
Lissencephaly - Clinodactyly - Polyhydramnios - Face	-
**3 markers**	
**Lissencephaly - Clinodactyly - Polyhydramnios**	-
**Lissencephaly - Clinodactyly** - **Face**	Microcephalic osteodysplastic primordial dwarfism
**Lissencephaly** - **Polyhydramnios - Face**	Neu-Laxova Sd
**Clinodactyly - Polyhydramnios - Face**	Rubinstein-Taybi Sd
**2 markers**	
Lissencephaly - Clinodactyly	Microcephalic osteodysplastic primordial dwarfism
Lissencephaly - Polyhydramnios	Neu-Laxova Sd
Lissencephaly - Face	5 other syndromes
Clinodactyly - Polyhydramnios	Rubinstein-Taybi Sd
Clinodactyly - Face	16 other syndromes
Polyhydramnios - Face	44 other syndromes

In addition, the database can offer guidance to the sonographer to find additional markers that differentiate between syndromes or genetic anomalies.

As this is a continuously evolving database (syndromes are being added on a daily basis), formal validation of sensitivity and specificity with validation against postnatal diagnosis has not been undertaken yet.

In order to test our database, all “cases of the week” from TheFetus.net were considered (380 cases, http://sonoworld.com/TheFetus/Listing.aspx?Id=2). Inclusion criteria were syndromes only. Exclusion criteria were cases with a unique organ involved (mainly bone and heart). All the remaining cases were considered (n = 50, see Table 2). Only prenatal markers based on ultrasound images from the Fetus.net website were used in Phenotip. In 12 cases (24%), one of the markers used was only present in the prenatal period (such as hydramnios, single umbilical artery). Among 50 unselected cases, 43 (86%) were found as correct and unique diagnosis. In 7 cases (14%), 2-5 diagnoses were identified, always including the correct diagnosis. By using all the data provided in the Fetus.net (karyotype, recurrence), many of these multiple diagnoses can be excluded. We are currently adding new Phenotip functions such as “known karyotype” and “previous case in the family” to increase specificity (Table 2).

**Table 2:** **Diagnosis found using prenatal images and corresponding markers from the Fetus.net**

**Cases**	**Ultrasound markers in Fetus.net**	**Diagnosis provided in Fetus.net**	**Phenotip diagnosis**
380	Macrocephaly, short long bones, polyhydramnios, platyspondily	Schneckenbecken dysplasia	same diagnosis
378	Hydrops, micromelia, ribs, narrow thorax, calcification of liver, polydactyly of hands	Greenberg dysplasia	same diagnosis
376	Mega cisterna magna, micrognathia, pulmonary valve stenosis	DiGeorge sd	2 diagnosis including the correct one^(1)^
370	Ventriculomegaly, craniosynostosis, prominent forehead, midfacial hypoplasia, macroglossia, renal cyst	Pfeiffer sd type II	same diagnosis
366	Macrocephaly, abnormal profile, polydactyly, sandal gap	Greig cephalopolysyndactyly	same diagnosis
363	Macrocephaly, hypoplastic thoracic cage, platyspondyly, micromelia, brachydactyly, bowed bones, low nasal bridge	Thanatophoric dysplasia type I	same diagnosis
361	Low nasal bridge, trident hands, frontal bossing, rhizomelia, narrow thorax	Achondronplasia	same diagnosis
357	Narrow thorax, bowed femurs, low set ears, clubfoot, nuchal edema, heart, retrognatia	Campomelic dysplasia	same diagnosis
345	Kyphoscoliosis, hemivertebra, ribs	Jarcho-Levin sd	same diagnosis
331	Ventriculomegaly, hypoplastic cerebellum, agyria	Walker-Warburg sd	same diagnosis
326	Flat nose, exophtalmia, cleft in soft palate, periventricular calcification, hypoplastic thoracic cage	Raine sd	same diagnosis
321	Cloverleaf shape, broad big toe, low nasal bridge, prominent eyes	Pfeiffer sd	same diagnosis
320	Accessory auricle	Goldenhar sd	3 diagnosis including the correct one^(2)^
318	Polydactyly of hands and feet, Rhizomelia/short femur and humerus, Ventricular septal defect	Ellis van Creveld sd	same diagnosis
316	Sacral agenesis, meningocele	Curranino sd	same diagnosis
314	Depressed nasal bridge, frontal bossing, mitten deformity, corpus callosum	Apert sd	same diagnosis
308	Hydrops, elbow pterygia, micrognathia	Multiple pterygium sd	same diagnosis
305	Polydactyly of hands, micromelia, hypoplastic thoracic cage	Short rib polydactyly	same diagnosis
302	Face, holoprosencephaly, anophtalmia, cleft lip	Cerebro-oculo-nasal sd	same diagnosis
290	Abnormal profile, hydramnios, single umbilical artery, micrognathia	Treacher Collins sd	same diagnosis
286	Soft tissu and bone hypertrophy, skin hemangiomas	Klippel-Trenaunay-Weber sd	same diagnosis
279	Postaxial polydactyly of toes, ascites, hydrometrocolpos	McKusick-Kaufman sd	same diagnosis
277	Skin, corpus callosum, cleft of soft palate	Pai sd	same diagnosis
272	Hydramnios, micromelia, narrow thorax, short ribs, hepatomegaly	Caffey disease	same diagnosis
263	Rhizomelia/short femur /short humerus, postaxial polydactyly, ASD, Hypoplastic thoracic cage	Ellis-Van Creveld sd	same diagnosis
257	Hydrops, barrel shape chest, omphalocele, micromelia	Achondrogenesis type I	same diagnosis
153	Kyphoscoliosis, neural tube defect, ventriculomegaly	Jarcho-Levin sd	same diagnosis
118	Polyhydramnios, small/collapsed stomach, (previous hepatomegaly & IUD)	Gaucher type II	4 diagnosis including the correct one^(3)^
117	Micrognathia, Mesomelia forearms, Hypoplastic thumbs	Nager sd	same diagnosis
100	Hydramnios, akinesia, talipes, face, hands	Myotonic dystrophy	same diagnosis
93	Cloverleaf skull, vertebral body, broad big toes, broad thumbs, prominent eyes	Pfeiffer sd type II	same diagnosis
81	Cloverleaf skull, micromelia, hydrocephalus, exophtalmia, hypoplastic thorax	Thanatophoric dysplasia II	same diagnosis
79	Abdominal wall, ectopia cordis	Pentalogy of cantrell	same diagnosis
77	IUGR, generalized edema, single umbilical artery (SUA)	Monosomy X	same diagnosis
75	Hypertelorism, dandy walker, dilated aorta, pulmonary valve stenosis, rocker bottom foot, clinodactily, pectus excavatum, SUA	Trisomy 9	same diagnosis
71	Hypospadia, nasal bone hypoplasia, micrognathia	Trisomy 21	same diagnosis
67	Clubfoot, limbs, sacrum	Atelosteogenesis type II	same diagnosis
65	Coarctation of aorta, unilateral hypoplasia of cerebellum, hemangioma	PHACE association	same diagnosis
48	Thick placenta, IUGR, anhydramnios/oligohydramnios	Trisomy 16	2 diagnosis including the correct one^(4)^
44	IUGR, polyhydramnios, increased NT, kydneys, broad thumbs, short long bones	Rubinstein Taybi Syndrome	same diagnosis
41	Holoprosencephaly, pectus excavatum, clenched hands, akinesia	Holoprosencephaly-fetal akinesia sequence	same diagnosis
40	Micrognathia, skin	Goldenhar sd	5 diagnosis including the correct one^(5)^
38	Hydrocephalus, thin upper lip, mega cisterna magna, extremities	Fryns sd	5 diagnosis including the correct one^(6)^
34	Polyhydramnios, nuchal thickening, micrognathia, poor ossification of ribs, receding forehead	Cerebro-costo-mandibular sd	same diagnosis
31	Micrognatia, renal hypoplasia, IUGR	Wolf-Hirschhorn sd	same diagnosis
30	Skin hemangiomas, renal	Klippel Trenaunay Weber sd	same diagnosis
23	Choroid plexus cyst, limbs, clenched hands, overlapping fingers, clubfoot, nuchal thickening	Pena Shokeir sd	2 diagnosis including the correct one^(7)^
20	Short limbs, overlapping fingers, clinodactyly, hypoplastic kidneys, ventriculomegaly, heart	Smith Lemli Opitz sd	same diagnosis
12	Omphalocele, bladder extrophy, neural tube defect, clubfoot	OEIS complex	same diagnosis
1	Oligohydramnnios, heart, micrognathia, placenta, sandal gap	Triploidy	same diagnosis

^(1)^Trisomy 18, DiGeorge sd.

^(2)^Cat-eye sd, Goldenhar sd, Branchio-oto-renal sd.

^(3)^Trisomy 18, Gaucher type II, VACTERL, Pallister Killian, if hepatomegaly considered, only Gaucher Type II sd.

^(4)^Trisomy 16, Meckel-Gruber sd type I.

^(5)^Cornelia de Lange sd, Multiple pterygium sd, Goldenhar sd, Neu laxova sd, trisomy 9.

^(6)^Fryns sd, Trisomy 13-18-21, Joubert sd.

^(7)^Pena shokeir, Trisomy 18.

Comparison between Phenotip and post-natal diagnosis.

Finally, the database is already designed to incorporate the relative frequency of each marker in each specific syndrome, so in the future the search will have even greater specificity and will use a Bayesian approach.

## Conclusion

We here describe the development of a searchable database of fetal syndromes. In contrast to other (commercially) available databases, this database only relies on antenatally diagnosable markers and does not include often subtle, postnatal findings.

We feel that this database may help both more and less experienced sonographers, obstetricians, geneticists and fetal medicine specialists in reaching the diagnosis of a fetal syndrome antenatally. Indeed, medicine involves large amounts of data that usually have to be exploited jointly. Given the limitations of the human brain, complex mathematical algorithms or Bayesian networks [9], integrating all available information can obtain better diagnostic accuracy.

Computer assisted diagnosis is already put in clinical practice on a daily basis in other branches of obstetrics and gynecology. Examples of this include prenatal screening for trisomy 21 [10], outcome prediction of pregnancies of unknown location [11] and discriminating between benign and malignant ovarian masses [12].

This database certainly does not replace expert fetal care providers as it still requires the input of accurate findings and will often only generate a differential diagnosis, which then needs to be explored further. Moreover, dealing with computed knowledge and software as tools for diagnosis does not substitute communication skills and empathy when facing patients.

This project is a work in progress and the number of syndromes included in the database will be further updated. Future developments will include the addition of magnetic resonance imaging markers [13] as well as further refinements in the search engine to allow prioritisation based on incidence of syndromes and markers. Moreover, we will add postnatal findings and information to each syndrome.

We anticipate that the growing use of advanced technologies (such as chromosomal microarray [14] or exome sequencing [15]) for the prenatal diagnosis of genetic alterations that are associated with sonographic abnormalities will discover novel, currently unknown, syndromes. This will further enhance the linkage between specific sonographic findings and the concomitant genomic alteration. As data gathers, we will incorporate those novel syndromes and information into the database. With this database, we hope to facilitate antenatal diagnosis of fetal syndromes and improve patient care.

### Presentation information

These data were presented at:

- 32^sd^ International Fetal Medicine and Surgery Society (IFMSS), Jerusalem, Israel - May 19-24, 2013 (*oral presentation*)
- 13^th^ World Congress in Fetal Medicine, Fetal Medicine Foundation, Nice, France, June 29^th^ – July 3^rd^, 2014, (*poster presentation*)
